# Orthomorphic Correction for Facial Asymmetry in a Patient With Long-Standing Temporomandibular Joint Ankylosis: A Case Report

**DOI:** 10.7759/cureus.43160

**Published:** 2023-08-08

**Authors:** Murtaza M Contractor, B M Rudagi, Kalyani Bhate, Sonali Deshmukh

**Affiliations:** 1 Oral and Maxillofacial Surgery, Dr. D. Y. Patil Dental College & Hospital, Dr. D. Y. Patil Vidyapeeth, Pune, IND; 2 Orthodontics and Dentofacial Orthopaedics, Dr. D. Y. Patil Dental College & Hospital, Dr. D. Y. Patil Vidyapeeth, Pune, IND

**Keywords:** temporomandibular joint, orthomorphic correction, reconstructive surgery, aesthetic surgery, facial deformity, tmj ankylosis

## Abstract

This case report presents the successful management of a 28-year-old female patient with facial deformity resulting from long-standing temporomandibular joint (TMJ) ankylosis. The patient underwent orthomorphic correction using a stereolithographic model of the upper and lower jaw to aid in surgical planning. The surgery was performed under general anesthesia via an intraoral approach. Cancellous bone graft harvested from the anterior iliac crest was utilized to cover the bone defect caused by the orthomorphic rotation of the lower jaw. The patient experienced satisfactory postoperative healing, and a six-month follow-up revealed significant improvement in facial symmetry and function.

## Introduction

Facial asymmetry is a condition characterized by an imbalance in the size, shape, or position of the facial structures, resulting in an aesthetically displeasing appearance. It can be caused by various factors, including congenital anomalies, developmental abnormalities, trauma, and acquired conditions such as temporomandibular joint (TMJ) ankylosis. Facial asymmetry not only affects the physical appearance of an individual but can also have a significant impact on their self-esteem and overall quality of life [1].

TMJ ankylosis, the fusion of the condyle to the temporal bone, is a complex disorder that can lead to severe facial asymmetry. This condition restricts jaw movement, resulting in functional limitations such as difficulty eating, difficulty speaking, and maintaining oral hygiene. Additionally, TMJ ankylosis causes significant facial deformity, leading to an imbalance in the symmetry and harmony of the face [1].

Facial asymmetry resulting from TMJ ankylosis poses a unique challenge to clinicians, as it requires a comprehensive treatment approach that addresses both the functional and aesthetic aspects. Surgical intervention is often necessary to restore normal jaw function, improve facial symmetry, and enhance the patient's quality of life [2].

In cases where adequate mouth opening has been achieved through previous surgical interventions, the primary focus shifts toward correcting the persistent facial asymmetry. Orthomorphic surgery, a technique that involves rotational correction of the affected jaw, offers a targeted and effective solution in such scenarios. This procedure aims to realign and reposition the lower jaw to restore facial symmetry and harmony, resulting in a more pleasing aesthetic appearance [3].

By implementing orthomorphic surgery, the surgeon can address the specific rotational component of the facial asymmetry caused by TMJ ankylosis [3]. Advanced tools, such as stereolithographic (STL) models of the upper and lower jaw, enhance the precision and accuracy of surgical planning, facilitating optimal outcomes for the patient.

In this case report, we present the successful management of a 28-year-old female patient with facial deformity resulting from long-standing TMJ ankylosis. The patient underwent orthomorphic correction, aided by an STL model, to achieve improved facial symmetry and restore functional balance. The postoperative healing and siz-month follow-up results demonstrate the effectiveness of orthomorphic surgery in addressing facial asymmetry associated with TMJ ankylosis.

## Case presentation

**Figure 1:**
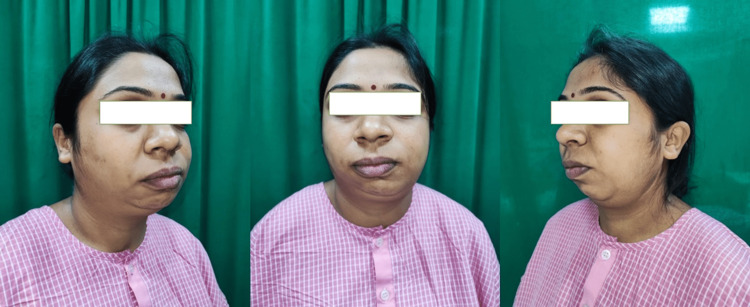
Pre-operative profile pictures.

A 28-year-old female patient presented with gross facial asymmetry due to a history of TMJ ankylosis. The patient had undergone a previous surgery for TMJ ankylosis five years ago, which resulted in a 38-mm interincisal distance but persistent facial deformity (Figure 1).

**Figure 2:**
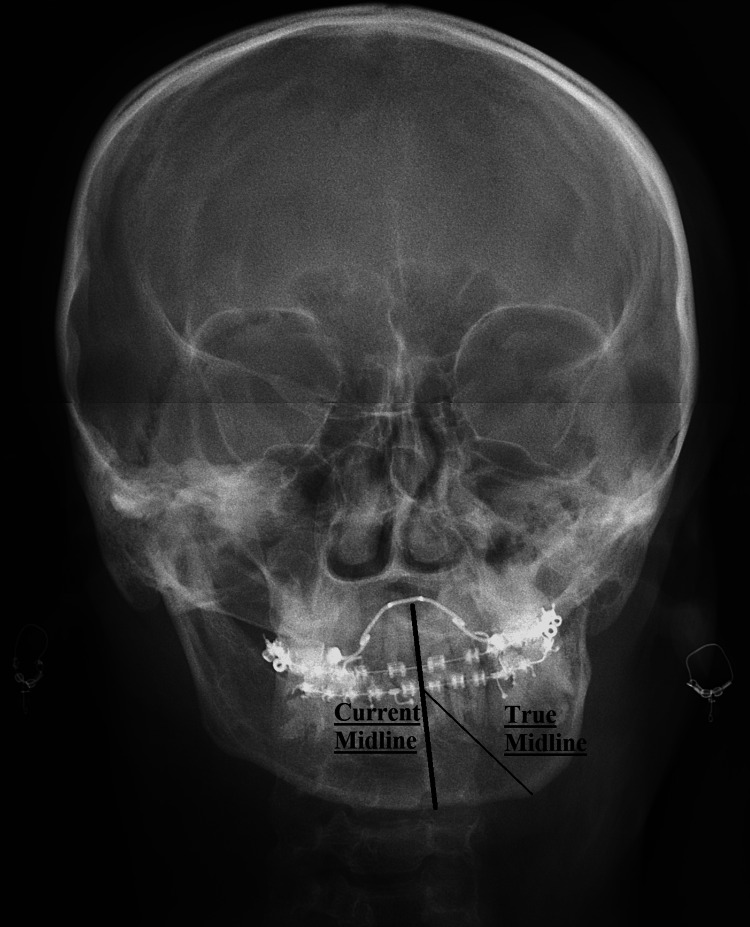
Pre-operative skull X-ray (posteroanterior view) showing shift of the true midline (point B: gnathion on cephalometry) toward the affected side on the mandible

Preoperative evaluation included a comprehensive assessment of the patient's dental occlusion, facial symmetry, and range of jaw movement (Figure 2).

**Figure 3:**
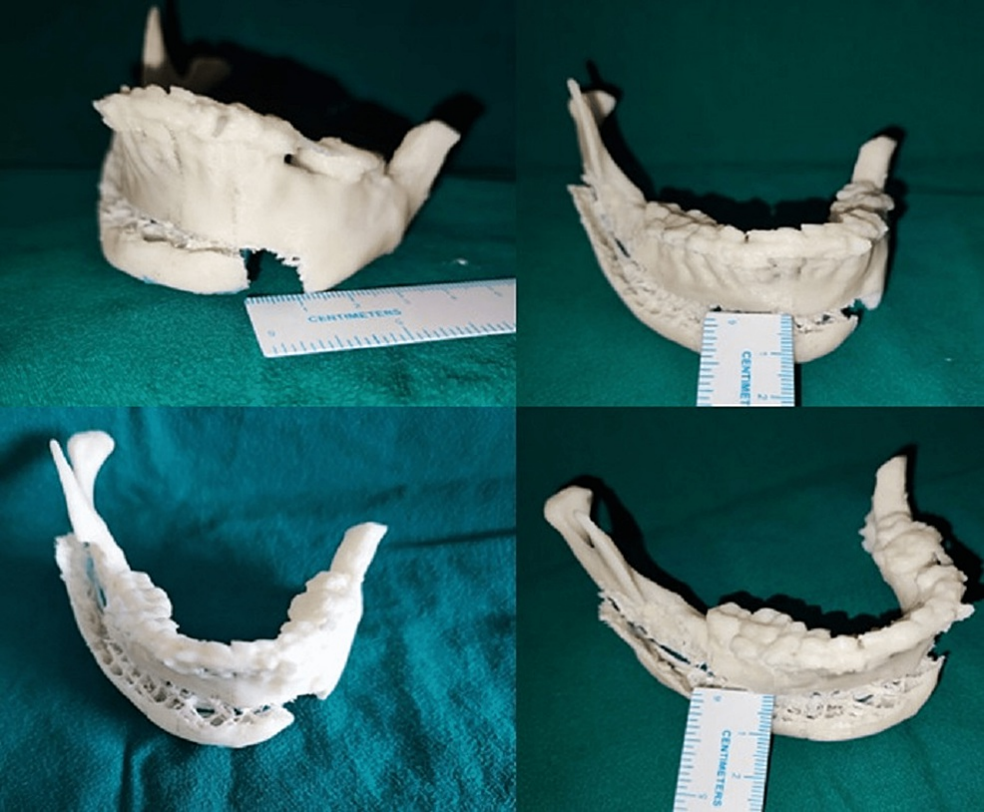
Surgical planning done on the stereolithographic model

An STL model of the upper and lower jaw was obtained to assist in surgical planning (Figure 3). Shift of midline 12 mm to medial and 7-mm advancement was done in STL model.

Surgical procedure

**Figure 4:**
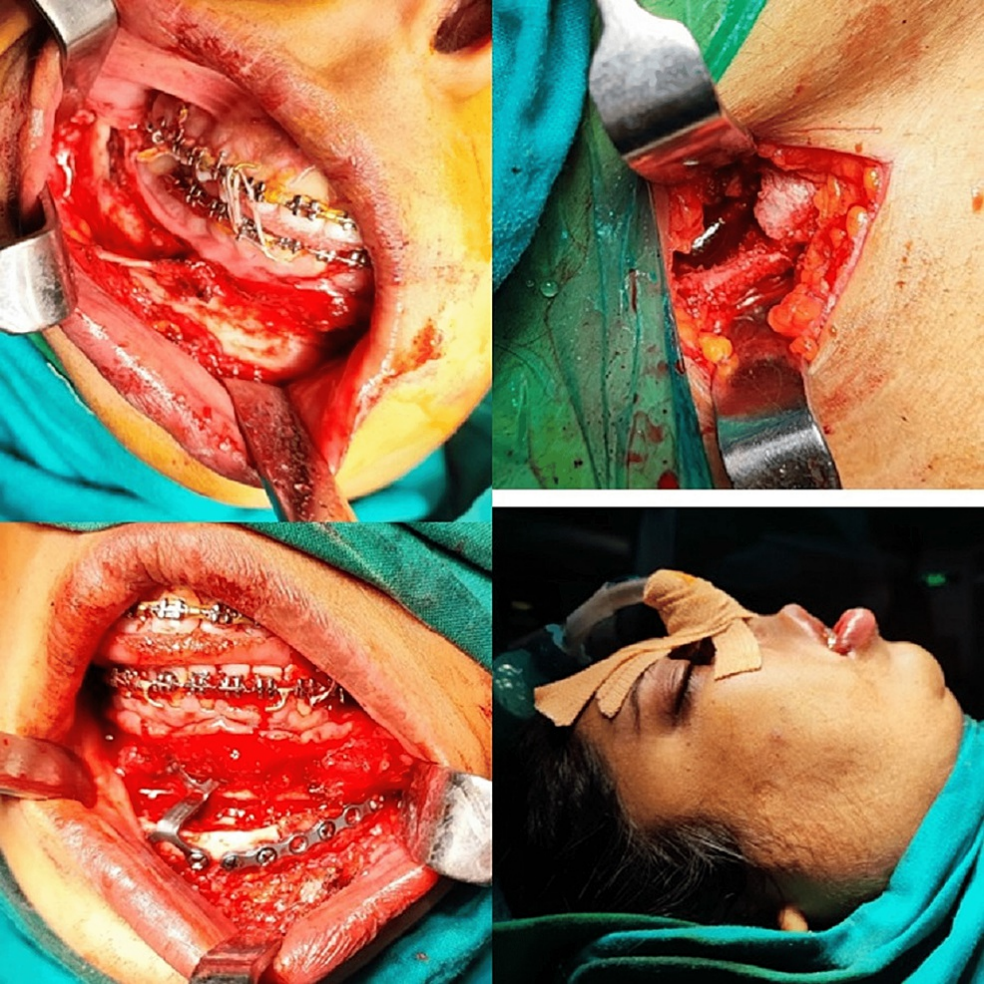
Intra-operative procedure pictures.

**Figure 5:**
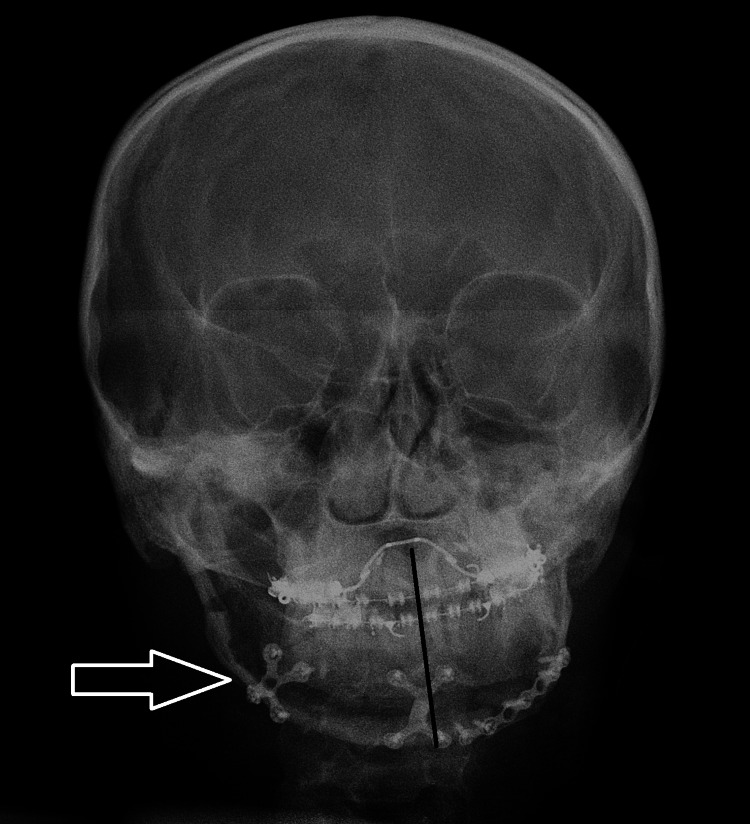
Immediate postoperative skull X-ray (posteroanterior view) showing correction in midline along with contour of the mandible (arrow).

The patient underwent orthomorphic correction under general anesthesia. An intraoral approach was employed for the surgical procedure. The surgical team performed an orthomorphic rotation of the lower jaw to achieve proper alignment and facial symmetry. To address the bone defect resulting from the rotation, cancellous bone graft harvested from the anterior iliac crest was used. Prior to harvesting the bone graft, the defect was measured for maintaining adequacy. The graft was carefully positioned and secured in place, ensuring optimal integration with the existing jawbone (Figures 4, 5).

Postoperative management

**Figure 6:**
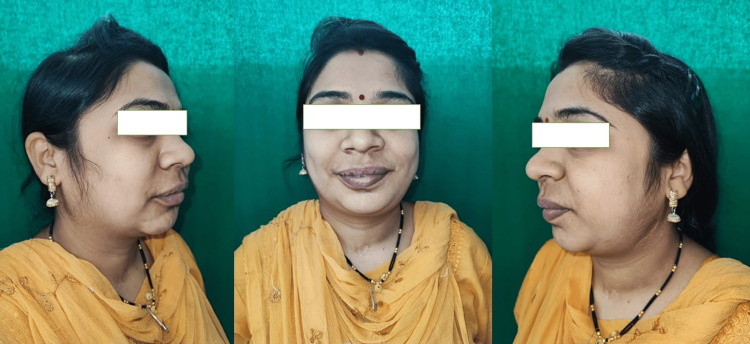
Six-month follow-up profile pictures.

Postoperatively, the patient was closely monitored for signs of complications and was prescribed appropriate pain management and antibiotics. Regular follow-up visits were scheduled to assess the healing process, functional outcomes, and patient satisfaction. At the six-month follow-up, the patient reported satisfactory results in terms of improved facial symmetry and function (Figure 6).

## Discussion

Orthomorphic surgery, also known as rotational orthognathic surgery or rotational correction, is a surgical technique used to correct facial asymmetry caused by various conditions, including TMJ ankylosis. It involves repositioning and rotating the affected jaw to achieve better alignment and restore facial harmony. The procedure aims to improve both aesthetic appearance and functional outcomes by addressing skeletal discrepancies.

In the case of TMJ ankylosis, orthomorphic surgery offers advantages over alternative approaches such as distraction osteogenesis or orthognathic surgery. Distraction osteogenesis involves gradually lengthening the bone through a distraction device to achieve desired jaw movement [4]. However, in cases of long-standing TMJ ankylosis with facial asymmetry, the flatness on the unaffected side requires additional bone grafting procedure, which may not be effectively addressed by distraction alone. Bilateral sagittal split osteotomy surgery, which is indicated for midline correction and asymmetry, was not possible in this case as the bone availability on the affected side was inadequate [5,6].

Orthomorphic surgery was chosen in this case because it offered a more straightforward and targeted correction of the patient's facial asymmetry resulting from long-standing TMJ ankylosis. The surgical plan was based on an STL model of the upper and lower jaw, which allowed for precise visualization and simulation of the rotational correction. By rotating the lower jaw, the procedure aimed to achieve facial symmetry and improve both functional and aesthetic outcomes.

TMJ ankylosis is a challenging condition that requires individualized treatment approaches. Orthomorphic correction is a viable surgical option for patients with adequate mouth opening but persistent facial deformity. The utilization of an STL model for surgical planning enhances precision and allows for better visualization of the corrective procedure. Harvesting cancellous bone graft from the anterior iliac crest offers a reliable source for reconstructing the bone defect caused by the orthomorphic rotation of the lower jaw. The satisfactory postoperative healing and favorable outcomes observed in this case highlight the effectiveness of orthomorphic correction in addressing facial asymmetry resulting from long-standing TMJ ankylosis.

## Conclusions

This case report demonstrates the successful management of facial deformity in a patient with long-standing TMJ ankylosis through orthomorphic correction. The utilization of an STL model for surgical planning and the use of cancellous bone graft contributed to satisfactory postoperative healing and improved facial aesthetics. Further research and large-scale studies are warranted to evaluate the long-term outcomes and determine the optimal surgical approaches for patients with TMJ ankylosis.
